# Widespread duplications in the genomes of laboratory stocks of *Dictyostelium discoideum*

**DOI:** 10.1186/gb-2008-9-4-r75

**Published:** 2008-04-22

**Authors:** Gareth Bloomfield, Yoshimasa Tanaka, Jason Skelton, Alasdair Ivens, Robert R Kay

**Affiliations:** 1 MRC Laboratory of Molecular Biology, Hills Road, Cambridge CB2 0QH, UK; 2 The Wellcome Trust Sanger Institute, Wellcome Trust Genome Campus, Hinxton, Cambridge CB10 1SA, UK

## Abstract

**Background:**

Duplications of stretches of the genome are an important source of individual genetic variation, but their unrecognized presence in laboratory organisms would be a confounding variable for genetic analysis.

**Results:**

We report here that duplications of 15 kb or more are common in the genome of the social amoeba *Dictyostelium discoideum*. Most stocks of the axenic 'workhorse' strains Ax2 and Ax3/4 obtained from different laboratories can be expected to carry different duplications. The auxotrophic strains DH1 and JH10 also bear previously unreported duplications. Strain Ax3/4 is known to carry a large duplication on chromosome 2 and this structure shows evidence of continuing instability; we find a further variable duplication on chromosome 5. These duplications are lacking in Ax2, which has instead a small duplication on chromosome 1. Stocks of the type isolate NC4 are similarly variable, though we have identified some approximating the assumed ancestral genotype. More recent wild-type isolates are almost without large duplications, but we can identify small deletions or regions of high divergence, possibly reflecting responses to local selective pressures. Duplications are scattered through most of the genome, and can be stable enough to reconstruct genealogies spanning decades of the history of the NC4 lineage. The expression level of many duplicated genes is increased with dosage, but for others it appears that some form of dosage compensation occurs.

**Conclusion:**

The genetic variation described here must underlie some of the phenotypic variation observed between strains from different laboratories. We suggest courses of action to alleviate the problem.

## Background

Genetic variation within a given species can extend from simple polymorphisms at single nucleotides to translocations, inversions and duplications affecting many genes. Recent work shows that such large-scale structural variation may be much more important than previously thought: for instance, the genomes of healthy human individuals may differ in copy number at hundreds of loci, that is, have distinct amplifications and deletions detectable by DNA microarray hybridizations [1-3]. These structural variations can have marked effects on phenotype as demonstrated by their association with pathologies of various kinds [4]. For instance, amplifications of alpha-synuclein cause a rare class of familial Parkinson's disease [5], and triplication of the trypsinogen locus can cause hereditary pancreatitis [6]. All sequence variation can, in principle, affect the function and regulation of genes and it is now possible to estimate the relative contribution of different kinds of mutation to changes in gene expression [7].

Similar variability can occur in laboratory organisms: inbred mouse strains show widespread copy number variation [8,9], which can be associated with complex phenotypes [10]. Budding yeast grown for generations in particular culture conditions displayed experimentally induced variations, reproducibly accumulating copy number mutations on certain chromosomes [11]; strains selected to suppress a loss of function mutation develop particular segmental duplications [12]. Spontaneous translocations have also been observed genetically in *Aspergillus nidulans *[13] and *Neurospora crassa *[14,15].

Copy number can influence phenotype through a proportional effect on mRNA abundance: aneuploidy, associated with direct increases in gene expression, is implicated in the antifungal drug resistance of certain *Candida albicans *strains [16]. These effects can also be of pathological significance: for instance, DNA copy number alteration is associated for many genes with altered gene expression in breast tumors [17,18] and progression of colorectal cancer coincides with large scale changes on copy number that are broadly mirrored by similar changes in mRNA level of affected genes [19].

*Dictyostelium discoideum *is a widely used laboratory organism, particularly useful for examining problems in cell biology, developmental signaling, the evolution of altruism and the function of conserved genes [20,21]. The organism grows as singled-celled amoebae, feeding on bacteria, and enters a multi-cellular stage when starved, to eventually produce a stalked fruiting body with a head of viable spores. Virtually all laboratory strains derive from the original type isolate from North Carolina, NC4, dating from 1933. Around 1970 two independent axenic strains - Ax2 and Ax3 - able to grow in complex media, were selected from NC4 [22,23]. These and their descendents now form the great majority of strains in current use.

*Dictyostelium *cells can be maintained as vegetatively growing amoebae or stored over long periods either frozen, or as spores. Although a sexual cycle via macrocyst formation exists, it has not been used as a laboratory tool [24,25]. Genetic exchanges are possible by a parasexual cycle, but are largely limited to chromosomal re-assortments with only a low frequency of recombination [26]. Today this cycle is not widely exploited. Most laboratory stocks therefore represent individual lineages that have become isolated from each other at various times in the past, and which may potentially have diverged from each other over time.

The published genome sequence of the Ax4 strain contains a large inverted segmental duplication on one chromosome [21,27], which is absent in other lines, notably the type strain NC4, from which Ax4 is ultimately derived. Other genetically marked strains have also been reported to contain duplicated chromosomes, or chromosome segments [28-30] and there are cases where duplicated genes are reported in particular stocks [31,32], but are only present as single copies in the sequenced genome. Pulsed field gel electrophoresis has also evidenced differences in chromosome size and number between certain strains [33].

These variations are of major practical importance to investigators, especially when they remain unknown, causing phenotypic differences between strains, and difficulties in genetic manipulation. We have surveyed a range of *Dictyostelium *laboratory strains and wild isolates using array comparative genomic hybridization and find that duplications are unfortunately widespread, such that the same strains, sourced from different laboratories, often differ substantially.

## Results

**Figure 1:**
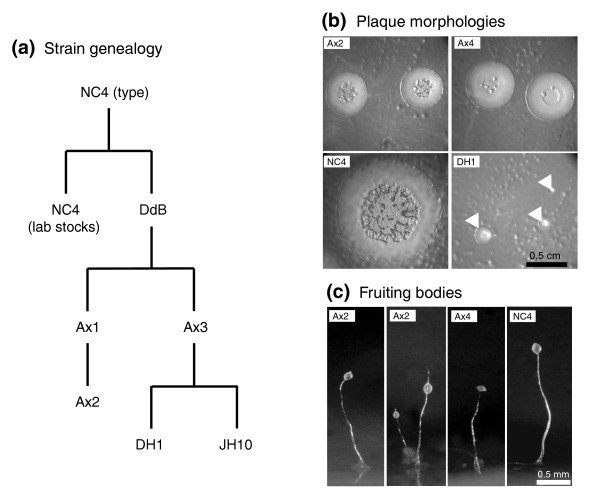
Relationships between the most commonly used *Dictyostelium *strains. **(a) **Simplified genealogical tree showing the relationships between common laboratory strains derived from NC4. The branch marked 'Ax3' is more complex than shown here: sub-lineages have been given the names KAx3 and Ax4. The auxotrophic strain DH1 was engineered in an 'Ax3' background, and JH10 from 'Ax4.' **(b) **Plaque morphologies. Cells were plated clonally in association with *Klebsiella aerogenes *on SM agar. Plaques were photographed after 4 days. Small DH1 plaques are indicated with arrowheads. Variation in diameter is a function of the rate of feeding and of the motility of the amoebae. Where the bacteria are cleared the amoebae aggregate in streams; this process had not yet begun in the slow-growing DH1 plaques. **(c) **Fruiting bodies. Wild type cells - in this instance NC4(Dee) - form larger, more robust fruiting bodies than axenic mutants.

Virtually all laboratory strains of *D. discoideum *derive from the original type isolate, NC4 [34], with only limited use being made of other wild isolates, such as V12. The axenic strains Ax2 and Ax3 are the most widely used and a particular lineage of Ax3, termed Ax4, has been fully sequenced [21]. A simplified family tree of this lineage is shown in Figure 1a. Axenic strains differ substantially from their parental NC4 stock: they grow more slowly on bacteria and produce smaller fruiting bodies, as is readily apparent from their plaque morphologies (Figure 1b,c). Amplifications and deletions (copy number variation) could be one source of this between-strain variability, in addition to small-scale mutation of individual genes and promoters.

To assess this potential source of variation, we used a custom-built DNA microarray to perform array comparative genome hybridization. In this procedure, DNA from a strain of interest and a reference strain is labeled with different dyes and the mixture hybridized to the array; after background subtraction the ratio of fluorescent signals gives the relative abundance of the DNA, which we normalize to 1 over the whole genome (log_2 _ratio of zero). Duplications should give a log_2 _ratio of 1 and deletions a large negative log_2 _ratio. In practice, cross-hybridization produces smaller than theoretically expected log_2 _ratios. Duplications can only be mapped to the nearest array marker, which average roughly 4 kb apart, and the procedure gives no information on chromosomal location of the duplication; their size is given as that of the region duplicated (thus the known duplication on chromosome 2 of Ax3 is referred to as 750 kb, not 1.5 Mb). The reference strain throughout was our version of Ax2 - called Ax2(Ka) - and other stocks were from the *Dictyostelium *Stock Center [35] or had been received into our laboratory in the past (Table 1).

**Table 1:** Strains used in this work

Strain	Donor	Stock centre strain ID	Reference
A2cycR	D Francis		[24,25]
Ax2-206	G Gerisch		
Ax2-214	G Gerisch		
Ax2(I)	R Insall		
Ax2(Ka)	RR Kay	DBS0235521	
Ax2(M)	D Malchow		
Ax2(Wee)	G Weeks (via SC)	DBS0235526	
Ax3(C)	R Chisholm (via SC)	DBS0235539	
Ax3(Dev)	P Devreotes (via SC)	DBS0235542	
Ax4(F)	R Firtel (via SC)	DBS0236487	
Ax4(Ku)	A Kuspa		
DdB(SC)	Stock Center	DBS0235747	
DdB(Wel)	D Welker		
DH1	P Devreotes		[37]
HU32	D Welker		[68]
JH10	R Firtel		[36]
KAx3(U)	H Urushihara		
NC28.2	D Francis		[46]
NC4(B)	J Bonner		
NC4(Dee)	R Deering (via D Welker)		
NC4(Kn)	D Knecht		
NC4(L)	W Loomis		
NC4(S)	P Schaap		
NC4(Wi)	K Williams (via D Welker)		
NC42.1	D Francis		[46]
NC4A2(Kn)	D Knecht		[44]
NC4A2(SC)	Stock Centre	DBS0236602	[44]
NC59.2	D Francis		[46]
NC66.2	D Francis		[46]
NC94.2	D Francis		[46]
NP73	D Welker		[69]
NP81	D Welker		[40]
NYA64	H Hagiwara		
V12M2	G Gerisch	DBS0235789	
WS205	D Francis		[24,25]
X22	P Newell		[41]
XP55	P Newell		[42]
XP99	P Newell		[43]

Most strains were chosen simply because stocks are held in this laboratory, having been previously sent for other purposes; others were obtained from the *Dictyostelium *Stock Centre [35]. Stock Centre strain IDs are given only where this is the exact strain tested - it was either deposited by us in the Stock Centre or received from it - but not otherwise.

### Duplications are frequent in laboratory stocks

**Figure 2:**
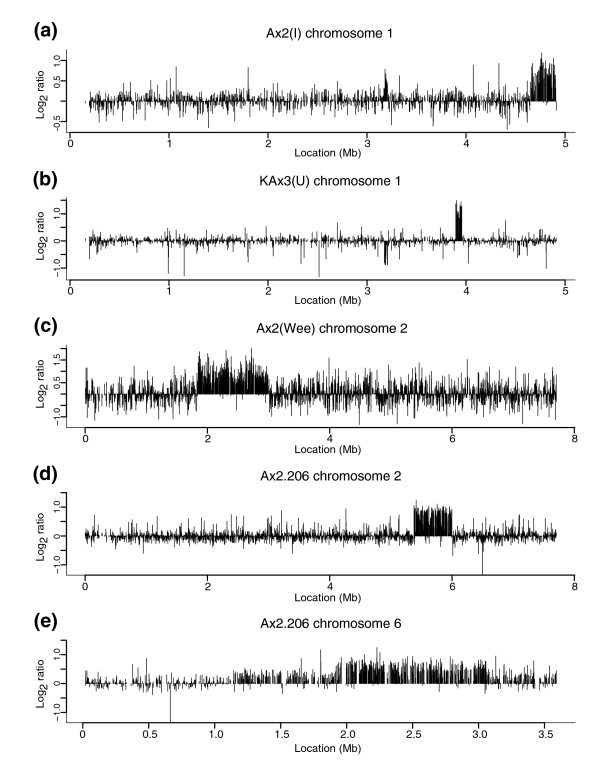
Duplications are frequent in 'wild type' axenic strains. **(a-e) **Log_2 _ratios (each strain compared to the Ax2(Ka) reference) are indicated by vertical lines; array probes are ordered according to their chromosomal location given by dictyBase assembly version 2.5. The previously known Ax3 duplication involves the region of chromosome 2 between approximately 2.25 and 3 Mb, which is wholly contained within the region duplicated in Ax2(Wee).

**Figure 3:**
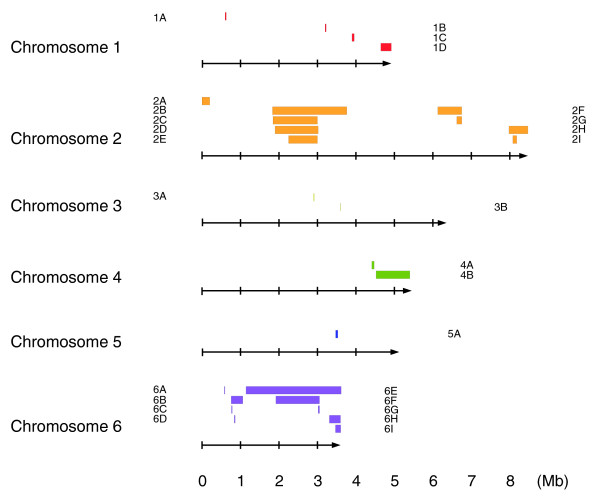
The distribution of amplifications across the genome. For each chromosome (depicted as arrows, with scale indicating Mb of sequence), different colored bars represent the segments duplicated, approximately to scale. Each feature is named according to the first column of Table 2, in which more precise data concerning size and location are given, along with the strains involved.

We examined 11 examples of the Ax2, Ax3, and Ax4 axenic strains. As expected, all Ax3/4 strains share the known chromosome 2 duplication (Figure S1 in Additional data file 1) and we also identified a small duplication/amplification on chromosome 1, common to all Ax2 strains, as described below. Apart from this, 9 of the 11 strains possessed additional duplications, some of which are shared between several lines, indicating clear patterns of relationship. Selected duplications are shown in Figure 2; the sizes and locations of all are given in Table 2, and chromosomal locations are displayed schematically in Figure 3.

**Table 2:** Chromosomal locations of duplications and their distribution among strains

Duplication	Chromosome	Start (gene)	Start (position)	Stop (gene)	Stop (position)	Length, bp (estimated)	Strain
1A	1	DDB0216544	597,838	DDB0202121	630,646	32,808	NP81, HU32
1B	1	DDB0190413	3,180,718	DDB0190424	3,207,169	26,451	Ax2(all)
1C	1	DDB0190683	3,902,919	DDB0190710	3,958,366	55,447	KAx3(U)
1D	1	DDB0190972	4,651,366	end	4,923,596	272,230	Ax2(I)
2A	2	end	1	DDB0216807	200,951	200,950	NP81
2B	2	DDB0217042	1,829,463	DDB0167938	3,760,461	1,930,998	Ax2(Wee)
2C	2	DDB0168867	1,848,568	DDB0217158	3,002,504	1,153,936	Ax2-214
2D	2	DDB0168894	1,898,568	DDB0231868	3,020,328	1,121,760	Ax2(M)
2E	2	DDB0185119	2,249,563	DDB0217157	3,002,134	752,571	Ax3/Ax4(all), NC4A2(both), JH10, DH1, HU32, NP81
2F	2	DDB0203552	6,131,391	DDB0217791	6,752,329	620,938	Ax2-206
2G	2	DDB0169405	6,623,914	DDB0217791	6,752,329	128,415	JH10
2H	2	DDB0217974	7,981,227	end	8,470,628	489,401	NC4(L), NC4(Kn)
2I	2	DDB0203385	8,080,299	DDB0217992	8,181,086	100,787	DH1, Ax3(D)
3A	3	DDB0206361	2,898,815	DDB0206368	2,915,972	17,157	NP81, HU32
3B	3	DDB0206089	3,595,775	DDB0206091	3,599,648	3,873	all non-NC4, some NC4s, X22
4A	4	DDB0186951	4,413,680	DDB0186970	4,474,299	60,619	NC28.2
4B	4	DDB0218826	4,572,845	end	5,450,249	877,404	NC4(B)
5A	5	DDB0219507	3,476,579	DDB0188678	3,531,501	54,922	DH1, Ax3(C), Ax3(D), Ax4(F), XP99, HU32, NP81
6A	6	DDB0183998	578,375	DDB0184007	595,296	16,921	NP81, HU32
6B	6	DDB0184069	763,797	DDB0184181	1,066,872	303,075	XP99
6C	6	DDB0219696	767,768	DDB0219699	787,282	19,514	NP81, HU32
6D	6	DDB0191606	838,926	DDB0184104	858,379	19,453	NP81, HU32
6E	6	DDB0184203	1,144,841	end	3,602,379	2,457,538	Ax2-206
6F	6	DDB0184511	1,919,891	DDB0219875	3,055,147	1,135,256	Ax2-206
6G	6	DDB0191998	3,022,031	DDB0219875	3,055,147	33,116	NP81, HU32
6H	6	DDB0192115	3,311,430	end	3,602,379	290,949	NC4(Wi)
6I	6	DDB0192193	3,468,862	end	3,602,379	133,517	NC4(S)

Breakpoints were estimated by eye, and their map locations determined by aligning the probe sequence with the dictyBase assembly version 2.5. The duplication in the sequenced strain is given as the breakpoints and size revealed by the sequence itself. As noted in the text there appears to be variation in this duplication among the different strains that inherited it. The putative duplications in Ax2-214 and Ax2(M) are atypical in that the average log_2 _ratio across their lengths is considerably lower than 0.5. The larger duplication of chromosome 6 sequence in Ax2-206 may be similar in this respect. We do not understand why these features differ from the more typical duplications we observe.

Four of the eleven strains carry unique duplications. Ax2(I) and KAx3(U) have duplications of parts of chromosome 1, of 274 and 62 kb, respectively (Figure 2a,b). Ax2(Wee) and Ax2-206 (a rarely used Ax2 clone from the Gerisch laboratory) bear larger 1,179 and 621 kb non-overlapping duplications from chromosome 2 (Figure 2c,d). The Ax2(Wee) duplication encompasses the Ax3 common duplication, plus around 400 kb to one side of it. This region is probably a hotspot, as three further, independent, duplications have been observed from expression profiling experiments comparing mutant with other strains (unpublished results). Ax2-206 also carries another large duplication of part of chromosome 6 (Figure 2e), within a larger region of log_2 _ratios greater than zero, but averaging less than we typically observe for regions present in two copies per genome. Ax2-214 (the standard Gerisch stock) and Ax2(M), ultimately deriving from the same laboratory, share a feature in the same region duplicated in Ax2(Wee) and Ax3 (Table 2). Log_2 _ratios in this feature are clearly shifted away from zero, but average less than 0.2. The basis of these 'sub-duplication' features is not known.

The auxotrophic mutant strains JH10 [36] and DH1 [37] - used as parental strains in molecular genetic studies - also show novel duplications. JH10 carries a unique 129 kb duplication of a segment of chromosome 2 (Table 2), while DH1 has two duplications, both shared with its parent Ax3(Dev) (Table 2, and below).

### Ax2 has a small duplication/amplification

**Figure 4:**
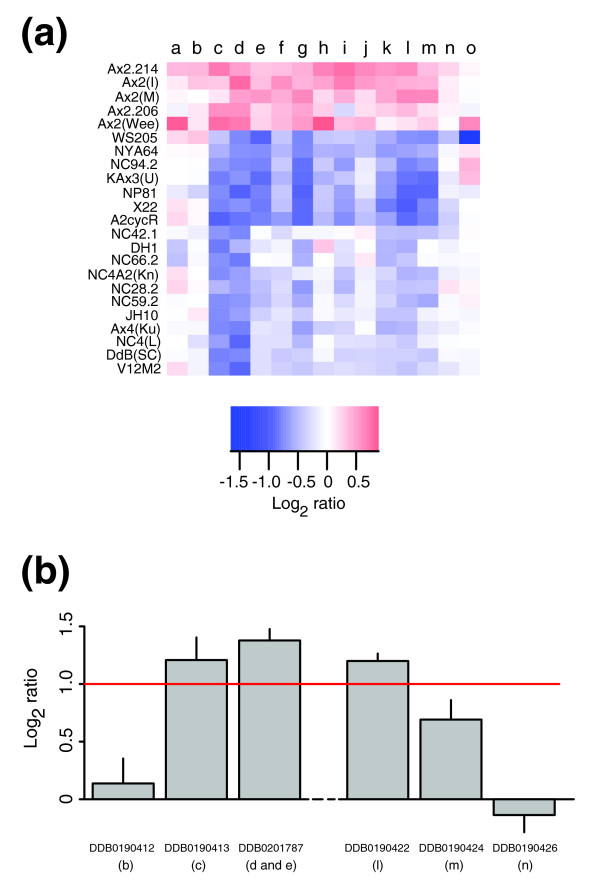
A duplication common to Ax2 lines. **(a) **All Ax2 strains in our study plus selected other strains of NC4 and non-NC4 backgrounds are displayed. Each block is colored according to the log_2 _ratio for the comparisons of each strain with reference Ax2(Ka). Since log_2 _ratios are consistently greater than zero for the duplicated genes in examples of Ax2 other than the reference, we suggest that this region is amplified further in these strains. The genes plotted are: a, DDB0190411; b, DDB0190412; c, DDB0190413; d, DDB0201787 (probe 1); e, DDB0201787 (probe 2); f, DDB0190415; g, DDB0190416; h, DDB0201789; i, DDB0190418; j, DDB0216669; k, DDB0190421; l, DDB0190422; m, DDB0190424; n, DDB0190426; and o, DDB0190427. **(b) **The breakpoints of the duplication in Ax2(Ka) were confirmed by real-time quantitative PCR, in comparison with Ax4(Ku). Mean log_2 _ratios ± standard error are shown, summarizing, per gene, four pairwise comparisons of threshold cycles.

A segment of 11 genes on chromosome 1 is under-represented in all strains tested compared to Ax2(Ka) (log_2 _ratio between -0.5 and -1) except for other examples of Ax2 (Figure 4a). This is most easily explained by an approximately 26 kb amplification common to all Ax2 lines, which presumably occurred when the original strain was selected, analogous to the much larger Ax3 duplication. Ax2(Ka) appears to have two copies of this sequence, but all the other Ax2s tested show an increase compared to Ax2(Ka), indicating three or more copies. The approximate breakpoints of this feature were confirmed by quantitative real-time PCR (Figure 4b). The genes amplified in Ax2 strains are listed in Table S1 in Additional data file 4; notably, there are three protein kinases, as well as a formin and a potential transcription factor.

### A segment of chromosome 5 is often duplicated in the Ax3 lineage

**Figure 5:**
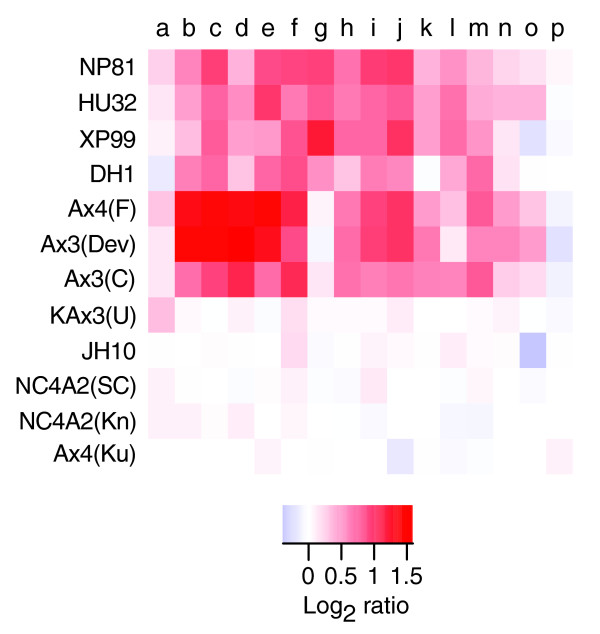
A novel duplication present in a subset of the Ax3 lineage. Nine strains in our study are lineal descendants of Ax3, and one other carries one or more chromosomes from it. NC4A2, based on our evidence, also descends from Ax3. Of these 12 lines, 7 carry a near identical duplication of chromosome 5 sequence. The breakpoints are not entirely clear because of noise in the data, and it is possible that there is some difference between strains. The genes plotted here are: a, DDB0188657; b, DDB0219507; c, DDB0188659; d, DDB0188660; e, DDB0188661; f, DDB0188665; g, DDB0188667; h, DDB0216146; i, DDB0188671; j, DDB0188673; k, DDB0188674; l, DDB0188677; m, DDB0188678; n, DDB0188686; o, DDB0188687; and p, DDB0188688.

Seven strains descending from Ax3 share a small duplication of chromosome 5 sequence, including Ax3(Dev) and its offspring DH1, as mentioned above (Figure 5). The duplicated genes are listed in Table S2 in Additional data file 4. Also among this group are the parasexually derived strains XP99, NP81, and the latter's offspring HU32, which all derive some, but not all, of their chromosomes from Ax3 (Figure S2 in Additional data file 2). Curiously, this feature is present in Ax4(F) but absent in that strain's presumed offspring JH10, though these two strains are clearly related because they share a three gene deletion not observed in any other strain (see below). The chromosome 5 duplication is also absent in several other examples of the Ax3 lineage, notably Ax4(Ku). It seems that this duplication must have arisen in the Ax3 lineage, but is relatively unstable, and has been independently lost at least twice (this seems a more likely explanation than the possibility of separate duplication events in, and only in, the Ax3 lineage).

### Strains used in parasexual genetics

Haploid *Dictyostelium *cells occasionally fuse to make fairly stable diploids, which can break down by random chromosome loss to reform haploids with a re-assorted chromosome complement. By selecting for diploid formation and breakdown, a workable parasexual system was developed for complementation testing and assigning markers to linkage groups [38]. However, this system sometimes produced anomalous results, to which unrecognized duplications might have contributed [39]. We therefore examined a number of strains dating from this parasexual era.

The most complicated pattern we have seen is given by NP81 and its offspring HU32. As well as multiple duplications, they also possess many contiguous regions of apparent gene loss (an example chromosome of each strain is shown in Figure S3 in Additional data file 3; all chromosomes show some stretches of gene loss). The log_2 _ratios in these regions are not extreme enough to suggest complete absence of the sequences, and in any case this is unlikely, given the likely presence of essential genes in these regions. They cannot represent duplications in the reference genome because the same DNA sample was used as reference in all hybridizations. We tentatively propose that these strains are degenerate diploids, hemizygous at the regions of apparent gene loss.

NP81 was selected for growth in the presence of the DNA damaging agent ethidium bromide [40] so it is not entirely surprising for its genome to show multiple abnormalities. In contrast, none of X22 [41], XP55 [42] and XP99 [43], which are derived from heavily mutagenized strains but not selected on ethidium bromide, show aberrations similar to NP81. There are no duplications discernible using our arrays in XP55 and X22, although XP99 has a unique one involving chromosome 6, as well as the smaller chromosome 5 feature it inherited from Ax3. The data for XP55 and X22 suggest that the once-standard methods of chemical mutagenesis and parasexual manipulation do not necessarily induce duplications at high frequency.

### NC4A2 carries a duplication indistinguishable from the chromosome 2 duplication common to all Ax3 strains

**Figure 6:**
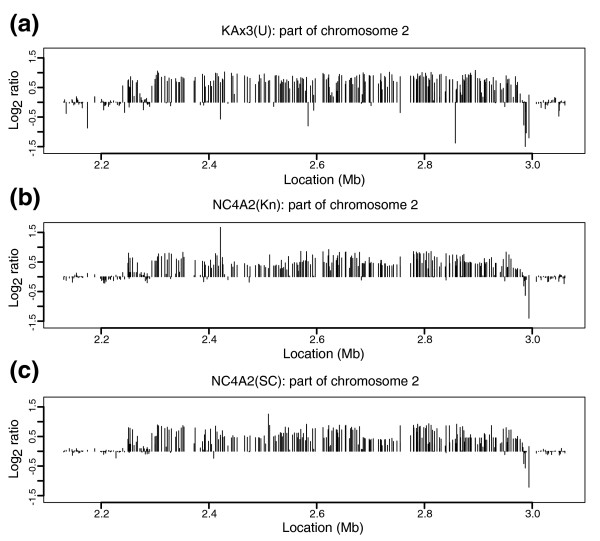
NC4A2 lines contain a duplication of the same segment of chromosome 2 that is duplicated in Ax3. The duplication appears for the most part identical in all strains derived from Ax3. We show here **(a) **Kax3(U), **(b) **NC4A2(Kn), and **(c) **NC4A2(SC) because they display points of similarity not observed in the other examples of this lineage in our study. The point of inversion of this tandem inverse duplication is to the right of the plot, where some genes (log_2 _ratios negative) appear to have been deleted in both copies in NC4A2 and KAx3(U). At least one of these genes appears to have been lost in both copies in several other of the Ax3-lineage strains in our study, but unfortunately some of the probes for these genes were not printed well and so our data do not permit us to assess exactly how frequent these deletions are. A segment within the duplication towards the left-hand side appears to be present as a single copy in both NC4A2 lines and in KAx3(U); this runs from DDB0233427 to DDB0191242, and appears to be present in the expected two copies in all other Ax3 derived strains we have studied.

NC4A2 is an axenic strain claimed to be directly selected from NC4, and in consequence, to have superior properties to the standard axenic strains [44]. However, two examples of this strain, obtained from different sources, both carry what appears to be the same chromosome 2 duplication as seen in Ax3 (Figure 6). Although regions of chromosome 2 have been duplicated independently several times, the breakpoints in this case are very similar (or indeed, the same) to those in Ax3; NC4A2 also lacks two other distinct duplications present in its presumed parent, NC4(Kn), as listed in Table 2. Thus, we believe that the strain currently designated as NC4A2 arose from inadvertent contamination by Ax3/Ax4 cells. There have been reports that its properties differ significantly from Ax4 (R Insall, personal communication), but in our hands its growth on bacteria and fruiting body morphology are much more similar to Ax4 than NC4 (not shown).

NC4A2 appears to be most closely related to KAx3(U), since both these strains have lost a segment of about 29 kb from one half of the inverted duplication on chromosome 2, which is now present as a single copy, and lack the other, novel, Ax3 duplication of chromosome 5 sequence. These and several other strains of the Ax3 lineage appear to have completely lost sequence near the point of inversion of the chromosome 2 duplication. The open reading frame designated DDB0217158 [45] is especially unstable. This mirrored region could be a target for recombination, leading to excision of segments. It is possible that the sequence of this region in Ax4(Ku), although apparently more complete than in some of its relatives, has also degenerated in the same way, resulting in the complete loss of some of the ancestral sequence.

### Duplications are also frequent in different stocks of NC4

**Figure 7:**
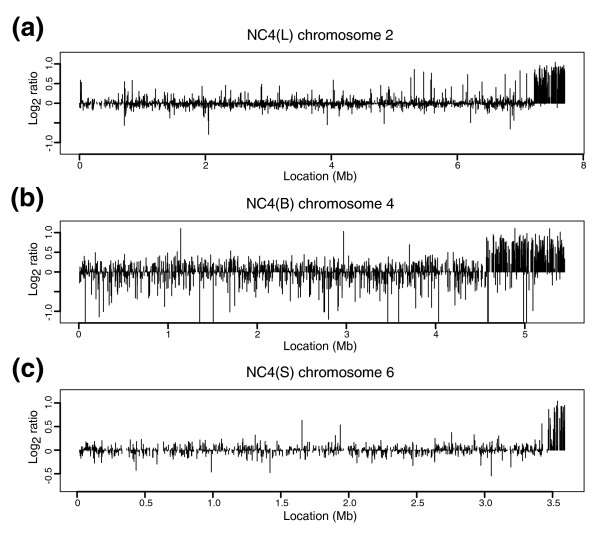
Duplications are also frequent in non-axenic wild types in the NC4 lineage. **(a) **NC4(L) chromosome (chr) 2, **(b) **NC4(B) chromosome 4, **(c) **NC4(S) chromosome 6. As well as the three strains shown, NC4(Wi) has a duplication overlapping that observed in NC4(S). It is not clear why duplications near the chromosome ends are especially frequent in these non-axenic lines, or indeed whether this is merely a sampling artifact. The parasexual segregant XP99 is not axenic and yet carries two duplications away from termini; however this strain inherited some of its chromosomes ultimately from Ax3 and we can be confident that one of these duplications occurred in that axenic ancestor.

To test whether duplications are a peculiarity of axenically maintained stocks, we examined a number of stocks of NC4, their non-axenic parent. We particularly sought lines of known history: for instance, NC4(S) came from a vial of spores lyophilized in the Raper laboratory in 1969, which was finally opened in the Schaap laboratory in 2005 (P Schaap, personal communication) and NC4(L) came directly from Raper, but was received in the Loomis laboratory after the generation of Ax3 (W Loomis, personal communication). We were surprised to find that most of the NC4 lines also contain duplications, which predominate in the sub-telomeric regions of the chromosomes (Figure 7 and Table 2). Again, most duplications differed in location in different lines, the exception being NC4(Kn), a stock of NC4(L) taken by D Knecht when he left the Loomis laboratory. This retains the same duplication as NC4(L), without gaining any further duplications, showing both that this duplication arose early and that duplications are not necessarily common. This duplication had been previously detected by 'mapping using haploid amounts of DNA and the polymerase chain reaction' (HAPPY mapping) - the strain is just referred to as NC4 in the paper [21] - but our estimate of its length at 495 kb is larger than the earlier rough estimate of 300 kb.

Since these duplications differ from stock to stock, we assumed that the original NC4 isolate lacked all of them, and therefore attempted to recover an NC4 strain of this genomic structure. Finally, we found three lines, DdB(SC), DdB(Wel), and NC4(Dee), which are without any discernible duplication, though they do lack a small duplication believed to be present in the founding NC4 stock (see below). DdB is a clone of NC4 that was selected in the laboratory of M Sussman, and NC4(Dee) was obtained by R Deering in the late 1960s from Sussman, then maintained in his laboratory, before transfer to D Welker in 1977 (D Welker, personal communication).

### Duplications in other wild isolates

The unexpected prevalence of duplications even among different stocks of NC4 might imply that the *Dictyostelium *genome is inherently unstable, or alternatively that instability is a consequence of laboratory culture. To examine this question we tested a number of other wild, little-cultured lines, including recent isolates made by D Francis at the site of the original type isolate at Little Butts Gap, North Carolina [46]. Only one of these seven strains shows evidence of a large duplication similar to those observed in laboratory strains (Table 2).

Two proximal derivatives of V12, another isolate from the wild that has been used as a standard non-axenic strain, were tested and found to be without such amplification: V12M2 is a clone of V12 chosen by G Gerisch and used for stalk cell inductions [47] and NP73 is an axenic derivative of V12 selected by K Williams (not shown). Two other wild strains, NYA64 and WS205, and a cycloheximide-resistant mutant derived from another wild isolate (A2cycR) also lack detectable duplications.

### Most wild isolates have a two-gene duplication that has been lost in all axenic strains

**Figure 8:**
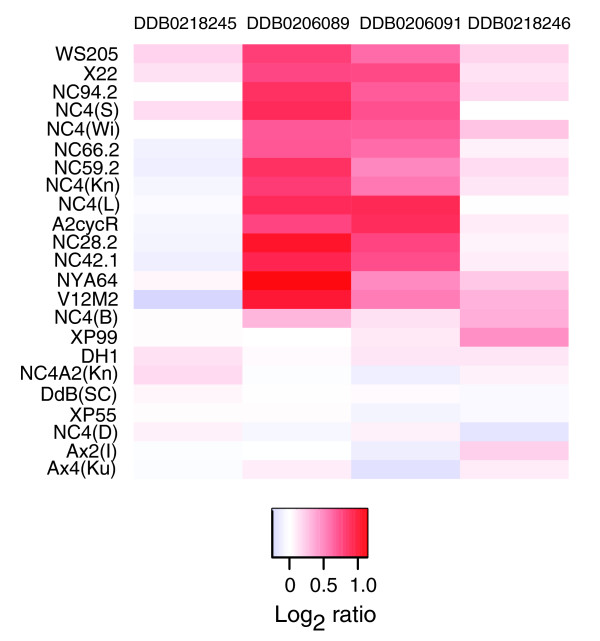
Two adjacent genes are duplicated in wild isolates but not in a subset of laboratory stocks, including all axenic strains. Shown here are all wild isolates in our study, plus selected laboratory strains descended from NC4. Note that some NC4 derived strains group with the wild strains, having two copies of these genes, but a subset (including all axenic strains) possess them in single copy.

Small duplications are difficult to distinguish from experimental noise at the level of replication used in this study. However, when present in a large enough portion of the sample they can still be reliably discerned. The notable example we found concerns two genes on chromosome 3. Remarkably, this duplication is found in all non-NC4 wild isolates tested (and A2cycR, a mutant derived from a Wisconsin wild isolate) and a subset of NC4 lines, including the mutant X22 (Figure 8). It is absent in NC4(Dee), the two DdB lines, NC4(B), two of the genetically marked non-axenic strains (XP55 and XP99), and all axenic lines tested. Note that the duplication is absent in NC4A2 but not its supposed parent NC4(Kn). Since it is extraordinarily unlikely that this clear division is the result of independent duplications in many different wild strains from different locations, we infer that this duplication was present in the original NC4 isolate, but a copy was lost in the DdB lineage of NC4 in the Sussman laboratory, and hence in all its descendants. The two duplicated genes encode a putative GATA transcription factor and a protein serine/threonine kinase; in between them is a small open reading frame that would encode a 46 amino acid polypeptide, if expressed.

### Deleted or diverged genes

**Figure 9:**
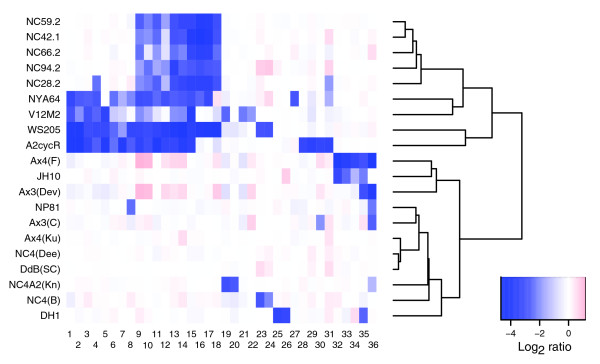
Potentially deleted sequences. Probes with extreme log_2 _ratios (below -3 in any strain) are considered to reflect deletions in that strain. The data have been clustered both by gene and by strain, to display relationships better (but information about groupings of genes along chromosomes is scrambled somewhat). The dendrogram gives an indication of relationships between strains, but note that this is based on the extremely small set of 36 genes shown here, and so should not be over-interpreted. The candidate genes are: 1, DDB0205403; 2, DDB0188003; 3, DDB0188004; 4, DDB0168894; 5, DDB0218478; 6, DDB0188007; 7, DDB0188002; 8, DDB0215073; 9, DDB0191949 (probe 1); 10, DDB0191949 (probe 2); 11, DDB0206115; 12, DDB0206106; 13, DDB0206110; 14, DDB0206111; 15, DDB0191930; 16, DDB0206108; 17, DDB0206112; 18, DDB0206109; 19, DDB0167672; 20, DDB0219404; 21, DDB0187848; 22, DDB0185937; 23, DDB0188514; 24, DDB0203140; 25, DDB0186442; 26, DDB0206404; 27, DDB0218143; 28, DDB0219338; 29, DDB0202734; 30, DDB0188991; 31, DDB0217456; 32, DDB0184376; 33, DDB0184375; 34, DDB219746; 35, DDB0206525; 36, DDB0217158.

Since essential genes are likely to be interspersed at regular intervals along all chromosomes, deletions are likely to encompass only a few genes and, therefore, will be more difficult to detect than large duplications. Potential deleted (or highly diverged) genes, compared to reference Ax2(Ka), were taken as those with a log_2 _ratio of less than -3. Their occurrence, in all wild isolates and in those laboratory strains in which they occur, is plotted in Figure 9. Of the very few such genes among NC4-derived strains, one is the engineered deletion of the *pyr5-6 *locus in the uridine auxotrophic strain DH1. Also notable is an apparent deletion on chromosome 6 shared by Ax4(F) and JH10 (which are from the same laboratory, with JH10 deriving from Ax4(F)). The three genes affected all show homology to known genes in other organisms.

By far the majority of these putative deletions are from wild isolates other than NC4. These strains tend also to have many measurements between log_2 _ratios -1 and -3, which likely represent a mixture of deletions and polymorphic sequences; the strain containing the most of these overall is NYA64, a Japanese isolate, and the only one not from the USA in our panel. Although these potentially deleted loci have not been confirmed by other means, they are listed in Table 3 as a resource for defining non-essential genes or (in the wild isolates) particularly divergent loci. None, other than *pyr5-6*, has been previously characterized well enough to appear in the published literature.

**Table 3:** Putative deleted genes

Gene ID	Annotation
DDB0167672	-
DDB0168894	bioY domain
DDB0184375	*pitD*: phosphatidylinositol transfer protein
DDB0184376	B-module
DDB0185937	Nucleotide binding protein 1-like protein
DDB0186442	*fslF*: frizzled/smoothened GPCR
DDB0187848	-
DDB0188002	-
DDB0188003	-
DDB0188004	-
DDB0188007	-
DDB0188514	-
DDB0188991	-
DDB0191930	-
DDB0191949	-
DDB0202734	Protein kinase related (catalytically inactive)
DDB0203140	-
DDB0205403	-
DDB0206106	-
DDB0206108	-
DDB0206109	-
DDB0206110	-
DDB0206111	-
DDB0206112	-
DDB0206115	*pyr5-6*
DDB0206404	-
DDB0206525	-
DDB0215073	-
DDB0217158	-
DDB0217456	-
DDB0218143	Protein kinase
DDB0218478	-
DDB0219338	-
DDB0219404	Dymeclin homologue - mutated in Dyggve-Melchior-Clausen syndrome
DDB0219746	-

Any gene with a log_2 _ratio below -3 in any comparison with the reference strain Ax2(Ka) is listed, along with any informative annotation available. The single gene deletion already known is *pyr5-6*, which was engineered in the creation of the auxotrophic strain DH1; this gene is not missing in any other strain. Strictly, some of these genes may merely have diverged in sequence in some strains sufficiently to significantly reduce hybridization to our probes (designed and amplified from the Ax4 genome).

### Effect of gene dosage on mRNA abundance

**Figure 10:**
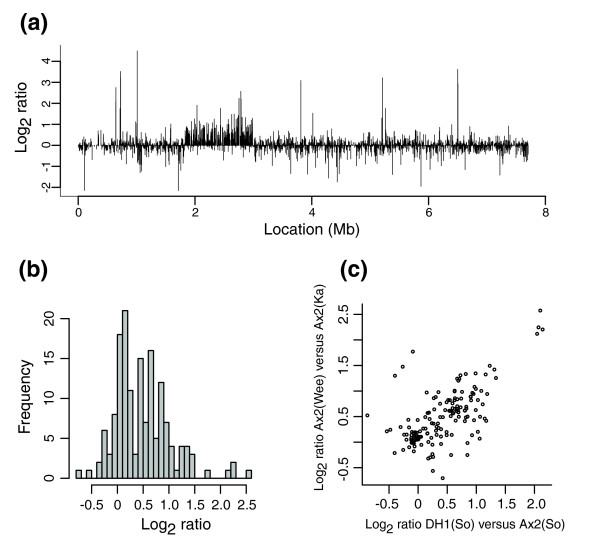
Duplications are apparent also at the mRNA level. **(a) **Data from an experiment comparing mRNA from growing Ax2(Ka) and Ax2(Wee) ordered by chromosomal location, for chromosome 2 only, where Ax2(Wee) has a duplication. This is the only region displaying a striking shift away from log_2 _ratio = 0. **(b) **Histogram of log_2 _ratios within the region duplicated in Ax2(Wee). The distribution tends towards bimodality, with one clear peak near log_2 _ratio = 0 and a less distinct one towards log_2 _ratio = 1. Thus, a portion of genes is dosage-insensitive. **(c) **Correlation between log_2 _ratios from independent mRNA comparisons. The duplication carried by Ax2(Wee) entirely encompasses the segment duplicated in Ax3 and its derivatives, allowing us to compare the dosage-sensitivity of genes in this overlap in different strains. The data are clearly correlated, with genes sensitive to dosage in Ax2(Wee) tending also to be sensitive in DH1 (unpublished work with T Soldati; the Soldati laboratory strains are indicated by So). A similar set of genes appears to undergo dosage compensation in both strains.

The most likely phenotypic consequence of gene duplication is through effects on the relative abundance of mRNAs expressed from genes within the duplication. To assess this we compared gene expression in growing Ax2(Wee) and Ax2(Ka) cells using the same microarray, and examined the relative expression of genes within the large duplication possessed by Ax2(Wee) on chromosome 2 (Figure 10a). In this case, gene dosage clearly affects the abundance of a large subset of vegetative mRNAs within the duplication, with some mRNAs being roughly twice as abundant as in the unduplicated parent, while many others are little altered (Figure 10b). The distribution of log_2 _ratios within the duplication is non-normal (Shapiro-Wilk test, *p *= 3.6 × 10^-6^). Since the amplified region in Ax2(Wee) includes the entire region duplicated in the Ax3 lineage, we could also compare these data with an expression experiment comparing DH1 with Ax2 (unpublished work with T Soldati). The log_2 _ratios of the two comparisons correlated well (Figure 10c), and a high proportion of the little-altered genes were shared between the two cases (Fisher's exact test, *p *= 2.7 × 10^-12 ^using a simple cut off of absolute log_2 _ratio less than 0.25; the *p*-value is similar if a cut off of 0.5 is used). Having excluded low intensity spots, and there being no obvious reason why these probes should have grossly underestimated differences in expression - for instance, there is very little correlation between mean log_2 _ratio and mean log intensity - we propose that, unlike budding yeast [12], *Dictyostelium *must possess modes of regulation that counteract increased gene dosage.

## Discussion

Our results reveal that duplications are common among all *Dictyostelium *laboratory stocks, except for those that have recently been isolated from the wild. This discovery brings an unwelcome element to phenotypic and molecular genetic experiments, but one that can be ameliorated by suitable precautions. We have also been able to glean useful information about *Dictyostelium *genealogy, identify some apparently non-essential genes, and loci that vary between wild-type stocks. Unexpectedly, our experiments also suggest that *Dictyostelium *has a form of gene-dosage compensation that acts on individual genes.

### Prevalence and practical consequences of duplications

The frequency of duplications is such that any two laboratory stocks of Ax2 or Ax3/4 or NC4 are more likely than not to differ by at least one duplication or related feature (with a conservative lower limit for detection of 30 kb). These differences range from unique duplications, estimated to occur in 30-40% of laboratory strains of the NC4 lineage, both axenic (5 out of 15 strains used as wild type, including DH1, JH10 and NC4A2) and non-axenic strains (3 of 8 strains of NC4 or DdB) to features common to several stocks of a strain, but differing in others, such as that on chromosome 1 of Ax2. We have not studied transformed strains systematically but in comparisons of mRNA levels between mutants and wild-types, novel duplications can be discerned in 5 out of 30 independent mutant strains (unpublished observations). This is likely to be a minimal estimate since the greater noise in RNA experiments means that smaller duplications will be missed. We have not examined any strains generated by restriction mediated integration (REMI) insertional mutagenesis [48].

The two most commonly used strains - Ax2 and Ax3/4 - were already known to differ by the large, 750 kb duplication on chromosome 2 present in all Ax3 derivatives, but we find two additional copy number differences between the lineages. Ax2 has a duplication of chromosome 1 sequence of about 26 kb and Ax3/4 has a second, less stable duplication on chromosome 5 of about 55 kb. This latter feature is present only in a subset of Ax3 derivatives, notably being absent from the sequenced strain Ax4(Ku), and JH10 despite being present in the latter's parent, Ax4(F). It was almost certainly present in the early history of Ax3, since it is found in strain XP99, which inherited chromosome 5 from NP2, which in turn was selected from Ax3 in the early 1970s [49].

The presence of duplications in the genome brings several experimental problems. The increased gene dosage causes increased RNA levels for at least some of the genes in the duplication, with unpredictable phenotypic consequences. Depending on the mechanism of duplication, there may also be damage at the event boundaries and the duplications themselves may be unstable, giving long-term genetic instability, as seen to some extent with the two duplications in the Ax3/4 lineage.

In addition to this, genetic manipulation of duplicated genes is much more problematic: two hits are required to knock them both out, which is achievable [50], but more difficult than when there is only a single locus to target. Duplicated regions would be expected to be relatively resistant to mutagenesis, since for recessive mutations, independent hits in both copies of the duplicated gene would be required to see the full phenotype. Effectively, this makes duplicated genes almost completely recalcitrant to genetic screens based on REMI insertional mutagenesis [48], which works at a frequency of roughly one hit per genome.

### Amelioration

It is clear that with good practice - strains stored as spores or frozen cells and working stocks renewed frequently - *Dictyostelium *stocks can be kept for many years without any detectable change. For instance, Ax2-214 stocks from the Gerisch and Malchow laboratories do not seem to have diverged despite a separation of more than 20 years, and nor does NC4(L) from the Loomis and Knecht laboratories. Although making a knock-out mutant may carry some risk of producing a duplication, the standard practice of comparing several independent clones, and rejecting any that are different, should greatly reduce the possibility of studying a phenotype that is due to secondary changes. Even so, it seems from the occurrence of duplications among knock-out stocks that it would be desirable to screen any new strain for copy number changes.

What is clearer than before is that it is risky to compare a mutant with anything other than its direct parent, or another mutant from the same parent. It was already known that the parental stocks Ax2 and Ax3/4 differ in fine detail when high-resolution phenotypic assays are applied, such as those for chemotaxis or patterns of developmental gene expression [51,52] (see the discussion of strain history at dictyBase for examples [53]), but it is now apparent that differences will exist even between stocks of the same strain. It would be advantageous in future to focus on just a few parental stocks, preferably those with a minimum of duplications, thus allowing wider phenotypic comparisons of mutants.

### Genealogy

Because the duplications and other features that we detect are quite stable over time, we can reconstruct some genealogies. The family tree of strains descended from the type isolate is given in Figure S2 of Additional data file 2. Our results broadly agree with the published histories of these strains, the one anomaly being NC4-A2 [44], which may originally have been an independent axenic isolate selected from NC4, but at some stage appears to have been contaminated and replaced by a line indistinguishable from Ax3.

We can also shed a little more light on the prehistory of the axenic strains. Most examples of NC4 and all the wild isolates we examined have a duplication of two genes on chromosome 3, which is missing in Ax2 and Ax3/4 and all their axenic derivatives - they have single copies of these two genes. Since Ax2 and Ax3 are of independent origin, they most likely share a common ancestor that also lacks the duplication. This is the case: strain DdB, their reported ancestor (see the discussion of strain history at dictyBase [45]; W Loomis, personal communication), has only single copies of these genes. DdB (also known as *D. discoideum *strain B, or NC4 strain B, not to be confused with the NC4(B) of this study) was a clone selected from NC4 in the laboratory of Maurice Sussman by 1967 at the latest [54,55]. Assuming that other strains lacking this small duplication also descend from the same clone, then members of the DdB lineage in our study include NC4 (Dee), which was obtained from the Sussman laboratory in 1968 or earlier [56]; the axenic strains NP81 and HU32, which inherited chromosome 3 ultimately from Ax3 [57]; XP55 and XP99, both of which inherited chromosome 3 (bearing the *bsgA *mutation) from NP194. NP194 is the offspring of NP20, a mutant that arose spontaneously from the DdB stock in the laboratory of P Newell [55].

We can only infer the original genomic structure of the NC4 type isolate. It should have lacked the sporadic duplications found in several existing stocks, such as NC4(S) and NC4(L), but had the small duplication on chromosome 3 discussed above. Unfortunately, we have been unable to recover an NC4 stock with exactly these characteristics. DdB, originating from the Sussman laboratory, may be the closest approximation, lacking only the small duplication.

### Mechanisms of duplication

Our method gives no information about duplication structure, and so we cannot even be sure that duplicates are on the same chromosome; experimental noise and the spacing of microarray probes also makes the precise determination of the breakpoints difficult. Both these factors limit our ability to identify possible mechanisms by which duplications arise.

However, the terminal position of several duplications suggests that these ones could have arisen from translocations. Some duplications appear to share one end to within a few kilobases, suggesting that certain regions are particularly prone to these events, presumably arising through the same mechanism. On chromosome two, duplications in JH10 and Ax2-206 both end near nucleotide 6,752,000, close to a complex repeat region associated with TRE3 repeats and two tRNA genes. The other breakpoint in Ax2-206 on this chromosome has a similar repeat. Therefore, a second possible mechanism is that the complex repeats found in scattered clusters throughout the genome [21,58] might promote unequal crossing-over events, thus duplicating segments of DNA between them [59]. Consistent with this, the Ax2(Wee) duplication is flanked by DIRS-1 sequences, and a further seven duplications have repeat sequences within 10 kb of one breakpoint (duplications 2G, 2I, 4B, 6A, 6D, 6F and 6H in Table 2). A subset of retrotransposons insert preferentially next to tRNA genes [58] and the chromosome six duplications in Ax2-206 and NP81 both end near nucleotide 3,055,000, which is near a tRNA gene; and another duplication in NP81 on the same chromosome (6A) is also flanked by tRNAs. It is possible that these strains have repeats inserted at the tRNAs that are not present in the Ax4(Ku) genome. For the other duplications, no complex repeats or tRNA genes could be identified near either breakpoint, so other mechanisms must be considered for these events. Detailed investigation of the breakpoint sequences will be required to address these issues.

We presume that most duplications become fixed when populations are established from single cells, although increased copy number of certain loci may also confer a selective advantage in laboratory conditions. Their rarity in the wild, as judged from recent isolates, suggests there may be negative selection pressures against duplications, confirming indirectly that they are likely to have phenotypic consequences. A genetically-inferred duplication on chromosome 3 was suggested to be detrimental to growth [29], so it is notable that the two duplications on this chromosome in the present study are very small, of approximately 4 and 17 kb in length.

### Deletions

Because essential genes are interspersed along all chromosomes, large deletions should be lethal, and indeed none are observed. A small number of NC4-derived strains have apparent deletions of at most three adjacent genes. This includes the engineered deletion of *pyr5-6 *in DH1 [37] but not of *thyA *in JH10, which is disrupted by an insertion rather than a deletion [36].

In wild isolates, apparent gene loss reflects a spectrum from sequence divergence to actual gene loss. More genes in wild isolates (other than NC4) pass our cutoff for putative deletions, but nevertheless the overall difference in gene repertoire is small, being at most 0.2% missing genes compared to NC4 (using a cutoff of log_2 _ratio < -3). As the array is effectively based on NC4, we cannot detect genes present in other isolates but not in NC4. Hints of geographical relationship are apparent: isolates from the type location cluster together most closely, and NYA64 from Japan (the only non-American isolate) is the most diverged when the log_2 _ratio cutoff is relaxed to allow detection of diverged sequence, rather than just those that are absent entirely.

### Gene dosage affects mRNA abundance, but not simply

In metazoans there is clear evidence that gene dosage effects feed through to the phenotype of the organism, so much so that compensatory mechanisms exist to counteract them. However, this may not be the case in yeast, as strains of budding yeast either aneuploid for entire chromosomes or carrying a smaller segmental duplication show little or no detectable dosage compensation [12]. We find that mRNA abundance is increased over a duplication, but varies from gene to gene, with some showing little increase. This variation does not appear to be due just to experimental noise, especially as there is a very good correlation between expression levels over two, independent, duplications spanning a common region. This might reflect incidental effects of differing modes of regulation of these genes - for instance, an activating factor could be limiting, or a repressor superabundant - but conceivably compensatory mechanisms to counteract dosage increase might exist for some genes, especially since duplication events seem to be so frequent.

## Conclusion

Our results show that previously unrecognized duplications are common in laboratory stocks of *D. discoideum*. These duplications are likely to alter the phenotype of the cells carrying them, and to cause problems, both when detailed phenotypes are examined, and in genetic manipulation. Nevertheless, stocks are relatively stable, and have been maintained over many years without apparent change, showing that with appropriate care, the problems caused by gratuitous duplications can be minimized. We also discerned possible genetic differences between wild isolates, and provide a list of potentially divergent loci.

## Materials and methods

*D. discoideum *strains were grown on SM agar plates in association with *Klebsiella aerogenes*, or else in axenic growth medium as previously described [60]. Strains were either obtained from the *Dictyostelium *Stock Centre or received into this laboratory as noted in Table 1. Different examples of the same strain are distinguished by letter codes indicating laboratories of origin. All strains were stored, without cloning, immediately upon receipt, either as spores (4°C or -20°C on silica gel with renewal times of 5-15 years) or frozen in liquid nitrogen (indefinitely viable).

To extract genomic DNA, cells were starved over-night and resuspended in lysis buffer (20 mM Tris-HCl, 5 mM MgCl_2_, 0.32 M sucrose, 0.02% sodium azide, 1% Triton X-100, pH 7.4) at 4°C, vortexed and incubated at 4°C for 15 minutes. Nuclei were pelleted at 3,000 g for 10 minutes, resuspended in lysis buffer and pelleted again, before freezing the pellets on dry ice. Proteinase K (100 μl (20 mg/ml)) was added, followed immediately by 10 ml digestion buffer (10 mM Tris-HCl, 5 mM EDTA, 0.7% EDTA, pH 7.5), and the pellet resuspended by gentle trituration. The lysate was incubated for 1 h at 60°C and the DNA phenol-chloroform extracted. DNA (5 μg) was labeled with Cy3 or Cy5 by incorporation of dye-conjugated dCTP (GE Healthcare, Little Chalfont, UK) from random hexamer primed strand synthesis catalyzed by the Klenow fragment (Invitrogen, Paisley, UK). Labeled samples were paired with an Ax2 (Ka) sample labeled with the complementary dye and hybridized to custom DNA microarrays printed on Codelink slides (GE Healthcare). Between two and four replicate hybridizations, always including both dye orientations, were carried out for each strain.

RNA was extracted using RNeasy kits (Qiagen, Crawley, UK). Samples were labeled and hybridized essentially similarly except that 25 μg of total RNA was labeled using anchored oligo(dT) primers and Superscript III reverse transcriptase (Invitrogen). Five independent replicates were carried out, two in one dye orientation, and three in the other.

The DNA microarray consisted of 9,247 PCR products non-redundantly representing 8,579 genes, with little bias regarding chromosomal location. The design was based on the sequence of strain Ax4(Ku), and genomic DNA of this strain was used as the template for PCR amplifications to generate the probes. The PCR products are predominantly between 200 and 400 bp in length (with a small fraction between 150 and 200 bp) and located towards the 3' end of predicted genes. Although overall coverage is good (8,579 genes non-redundantly represented of 10,500-12,500 predicted), non-coding DNA, including ncRNA genes, is not covered at all (unless accidentally), and some regions are more sparsely covered than others. Probes are ordered according to dictyBase assembly version 2.5 [45]).

Microarrays were hybridized for 16-18 h at 42°C, washed at room temperature, and scanned using a Genepix 4000B scanner (Molecular Devices, Wokingham, UK). Images were quantified using Genepix 3 software, then background-subtracted using the Kooperberg function, normalized with the print-tip loess algorithm, and model-fitted using Limma [61-63]. Data from low intensity probes (defined as mean log_2 _intensity in the hybridizations involving a particular strain less than 6) were omitted; in such a case the median was taken of the log_2 _ratios of the ten probes either side of it as they are arranged chromosomally (except those of mean log2 intensity less than 6). For probes within 10 of the end of a chromosome a median of the 20 terminal probes was taken (except those of mean log_2 _intensity less than 6).

The known 750 kb duplication of the sequenced strain Ax4(Ku) was readily apparent (Figure S1 in Additional data file 1), standing out on average 5.4 standard deviations beyond the remainder of chromosome 2 probes. The mean log_2 _ratio of probes within the duplication is 0.60. A similar underestimation relative to the expected value of one for a duplication in a haploid strain was observed for many different duplications, and presumably reflects a hybridization or scanning artifact, perhaps caused by cross-hybridization of equal copy-number probes. Noise in the data was approximately the same in hybridizations of the same DNA labeled in both channels as in comparisons of different strains (as assessed by overall standard deviation) and so can be explained mostly by experimental error, and not, for example, by sequence variation.

Novel duplications were identified by direct inspection of the data, and on occasion rough breakpoint positions were estimated using DNAcopy [64]. Complex repeat sequences were identified by performing BLAST alignments [65] of the 20 kb sequence surrounding our estimate of the breakpoint position against a database of the sequences described in [66].

Quantitative real-time PCR was carried out using Superscript III and Platinum Taq (Invitrogen) and an Mx3000P thermal cycler (Stratagene, Amsterdam, The Netherlands). Standard curves were made for each primer pair using Ax4(Ku) genomic DNA, and four comparisons made between that template and genomic DNA of Ax2(Ka). The mean of these four log_2 _ratios thus obtained was then taken.

Array data are deposited at Array Express [67] as array design A-SGRP-3 and the data as submission E-TABM-394.

## Abbreviations

REMI mutagenesis, REstriction Mediated Integration mutagenesis.

## Authors' contributions

GB, JS and AI designed and built the *Dictyostelium *microarray. GB, AI and RRK conceived of the study. GB, YT and RRK prepared biological material. GB, JS and YT performed microarray experiments. GB and JS analyzed the data. GB and RRK prepared the paper.

## Additional data files

The following additional data are available with the online version of this paper. Additional data file 1 is Figure S1 showing the Ax3/4 duplication on chromosome 2. Additional data file 2 is Figure S2 showing the genealogy of NC4-derived strains. Additional data file 3 is Figure S3, showing multiple copy number variants on chromosome 5 of strains NP81 and HU32. Additional data file 4 contains Table S1, listing genes present on the Ax2 duplication, Table S2, listing genes on the chromosome 5 duplication present in many Ax3 lines, and the legends for Figures S1, S2 and S3.

## Supplementary Material

Additional data file 1 The Ax3/4 duplication on chromosome 2. Click here for file

Additional data file 2 Genealogy of NC4-derived strains. Click here for file

Additional data file 3 Multiple copy number variants on chromosome 5 of strains NP81 and HU32. Click here for file

Additional data file 4 Genes present on the Ax2 duplication, genes on the chromosome 5 duplication present in many Ax3 lines, and legends for Figures S1, S2 and S3. Click here for file
