# Supplementary figures and images for: Widespread duplications in the genomes of laboratory stocks of Dictyostelium discoideum
Source: Genome Biol. 2008 Apr 22;9(4):R75. doi: 10.1186/gb-2008-9-4-r75 (PMC2643946; doi:10.1186/gb-2008-9-4-r75)

## Ax4(Ku) chr2

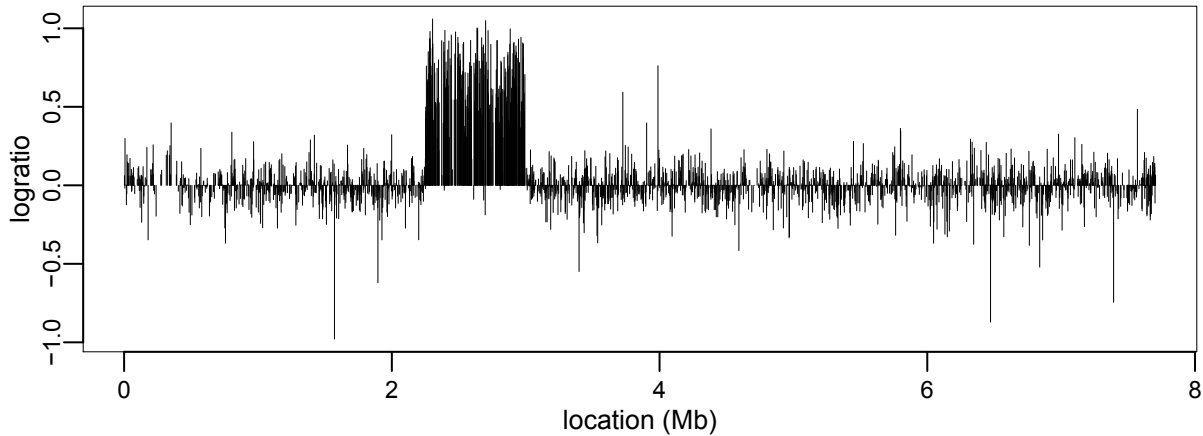

Supplement: Additional data file 1 — The Ax3/4 duplication on chromosome 2. [file gb-2008-9-4-r75-S1.pdf]

NC4 (Type)

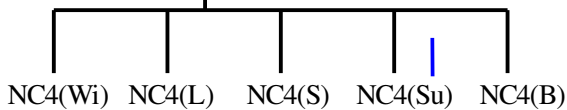

NC4 (Kn)

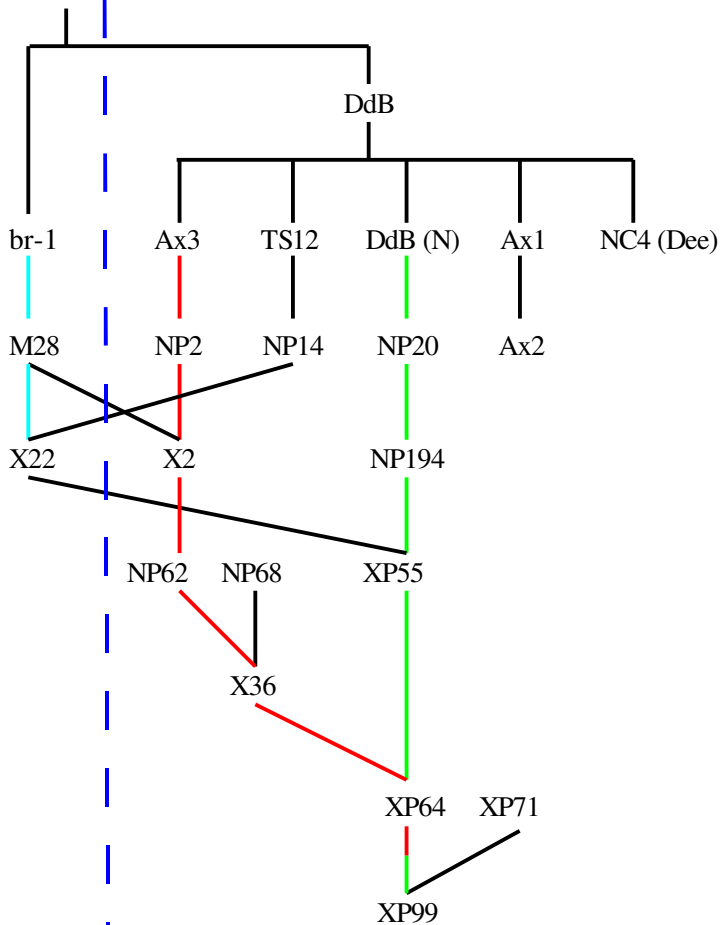

Supplement: Additional data file 2 — Genealogy of NC4-derived strains. [file gb-2008-9-4-r75-S2.pdf]

**A****NP81 chr5**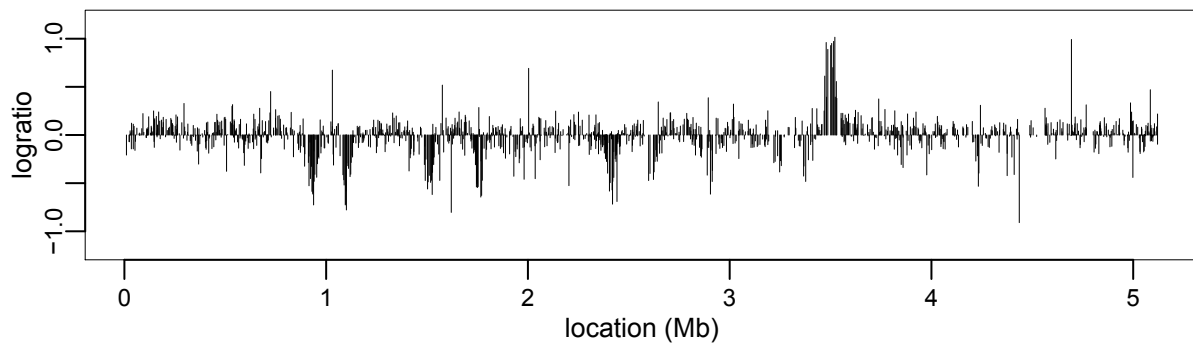**B****HU32 chr5**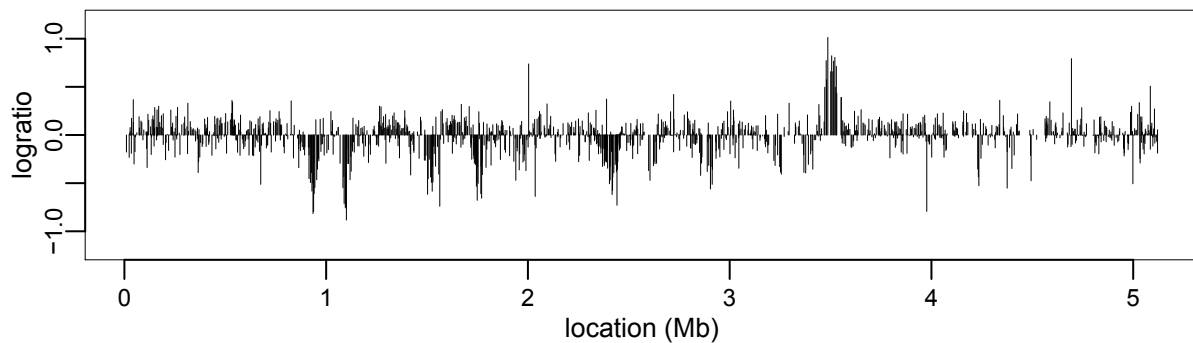

Supplement: Additional data file 3 — Multiple copy number variants on chromosome 5 of strains NP81 and HU32. [file gb-2008-9-4-r75-S3.pdf]
