# Supplementary material for: Widespread duplications in the genomes of laboratory stocks of Dictyostelium discoideum
Source: Genome Biol. 2008 Apr 22;9(4):R75. doi: 10.1186/gb-2008-9-4-r75 (PMC2643946; doi:10.1186/gb-2008-9-4-r75)
Supplement: Additional data file 4 — Genes present on the Ax2 duplication, genes on the chromosome 5 duplication present in many Ax3 lines, and legends for Figures S1, S2 and S3. [file gb-2008-9-4-r75-S4.pdf]

# Supplementary material

| Gene ID | Annotation |
|---|---|
| DDB0190413 | Formin: *forE* |
| DDB0201787 | Putative transport transmembrane protein |
| DDB0190415 | Acetyltransferase (GNAT) family + protein kinase domain |
| DDB0190416 | TatD related DNase domain |
| DDB0237678 | - |
| DDB0237324* | rnu2b – spliceosomal ncRNA gene |
| DDB0190418 | Protein kinase: *irlD* |
| DDB0216669 | Protein kinase: *irlC* |
| DDB0190420* | - |
| DDB0190421 | EF hand containing |
| DDB0190422 | Histone-like transcription factor |
| DDB0190423* | Contains KOW motif |
| DDB0190424 | Mak16l (involved in ribosome biosynthesis) |

**Table S1**. Genes present in the Ax2 duplication. Genes marked with an asterisk (*) do not have probes on our array.

| Gene ID | Annotation |
|---|---|
| DDB0219507 | - |
| DDB0188659 | RNA recognition motif |
| DDB0188660 | - |
| DDB0188661 | rps9 (40S ribosomal protein S9) |
| DDB0188662* | - |
| DDB0188663* | - |
| DDB0188664* | - |
| DDB0188665 | *sigF* |
| DDB0188666* | - |
| DDB0188667 | Cellulase domain |
| DDB0188669* | - |
| DDB0216146 | - |
| DDB0188671 | Pumilio-family RNA binding protein |
| DDB0215951* | - |
| DDB0188672* | - |
| DDB0188673 | - |
| DDB0188674 | Acyl-CoA oxidase |
| DDB0188675* | Contains FNIP repeat |
| DDB0188676* | - |
| DDB0188677 | - |
| DDB0188678 | - |
| DDB0219508* | - |
| DDB0219509* | - |
| DDB0188681* | - |
| DDB0188682* | *grxA*: glutaredoxin |
| DDB0215953* | - |
| DDB0188683* | DnaJ domain |
| DDB0188684* | - |
| DDB0188685* | - |
| DDB0188686 | Sepiapterin reductase |
| DDB0188687 | *gxcL* (RhoGEF domain) |

**Table S2.** Genes present in the chromosome 5 duplication found in many strains derived from Ax3. Genes marked with an asterisk (*) do not have probes on our array.

## Legends to supplementary figures

**Figure S1 (Additional data file 1)**. The Ax3/4 duplication on chromosome 2. The known duplication on chromosome 2 of the sequenced strain Ax4(Ku) is clearly apparent in comparison with our reference strain Ax2(Ka) at the level of genomic DNA. All probes for chromosome 2 genes are ordered according to dictyBase assembly version 2.5, and log2 ratios indicated by vertical lines.

**Figure S2 (Additional data file 2)**. Genealogy of NC4-derived strains, combining known relationships with those posited from our study. Strains beneath vertical lines alone arose by simple lineal descent of haploid lines, with or without selection and/or mutagenesis; horizontal lines divide multiple lines of simple descent from the same strain (at different times); strains beneath conjoining vertical and diagonal lines arose through parasexual crosses: both haploid parents are indicated, but the diploid intermediate is omitted for clarity. The dashed blue vertical line divides strains that possess the 2-gene duplication on chromosome 3 (left) from those that lack it (right). We assume that strain DdB was selected in the Sussman laboratory after the isolation of the brown mutant br-1, which was relatively early [70,71]. X22 descends both from br-1 and TS12, so we infer that it inherited its copy of chromosome 3 from the former (indicated by cyan lines). XP55 and its descendent XP99 descend in part from X22 [43] but lack the duplication. This is expected because they carry the *bsgA* mutation on that chromosome, inherited from NP194, which descends from the Newell laboratory stock of DdB [42,55]. This line of descent is shown in green. XP99 descends in part from Ax3, and possesses a duplication of chromosome 5 sequence common in derivatives of it. We can trace chromosome 5 of XP99 back to strain X2, because its offspring NP62 gained the *cobA* mutation on this chromosome [43]. We assume that X2 inherited chromosome 5 from Ax3 via NP2; this line to XP99 is marked in red. The relationship of NC4(B) with other strains is uncertain.

**Figure S3 (Additional data file 3)**. NP81 and HU32 show multiple copy number variants. Chromosome 5 data are shown for both strains; all other chromosomes also show apparent losses relative to the reference strain, and duplications observed are

listed in Table 2.  The single clear duplication on chromosome 5 shown here is the one also found in many Ax3 descendents.
